# Publisher Correction: Self-association of MreC as a regulatory signal in bacterial cell wall elongation

**DOI:** 10.1038/s41467-022-28008-1

**Published:** 2022-01-11

**Authors:** Alexandre Martins, Carlos Contreras-Martel, Manon Janet-Maitre, Mayara M. Miyachiro, Leandro F. Estrozi, Daniel Maragno Trindade, Caíque C. Malospirito, Fernanda Rodrigues-Costa, Lionel Imbert, Viviana Job, Guy Schoehn, Ina Attrée, Andréa Dessen

**Affiliations:** 1 Univ. Grenoble Alpes, CEA, CNRS, Institut de Biologie Structurale (IBS), Grenoble, France; 2 Univ. Grenoble Alpes, CNRS ERL5261, CEA-IRIG-BCI, INSERM UMR1036, Grenoble, France; 3 Brazilian Biosciences National Laboratory (LNBio), CNPEM, Campinas, São Paulo, Brazil; 4 Departamento de Genética, Evolução, Microbiologia e Imunologia, Instituto de Biologia, Universidade Estadual de Campinas (UNICAMP), Campinas, São Paulo, Brazil

**Keywords:** Bacterial structural biology, Cryoelectron microscopy, X-ray crystallography

Correction to: *Nature Communications* 10.1038/s41467-021-22957-9, published online 20 May 2021.

In this article the caption to Fig 4 was inadvertently omitted. The original article has been corrected.

