# Supplementary material for: Self-association of MreC as a regulatory signal in bacterial cell wall elongation
Source: Nat Commun. 2021 May 20;12:2987. doi: 10.1038/s41467-021-22957-9 (PMC8137920; doi:10.1038/s41467-021-22957-9)
Supplement: Supplementary file 1 — Supplementary Information [file 41467_2021_22957_MOESM1_ESM.pdf]

SUPPLEMENTARY INFORMATION FOR

# Self-association of MreC as a regulatory signal in bacterial cell wall elongation

Alexandre Martins, Carlos Contreras-Martel, Manon Janet-Maitre, Mayara M. Miyachiro, Leandro F. Estrozi, Daniel Maragno Trindade, Caique C. Malospirito, Fernanda Rodrigues-Costa, Lionel Imbert, Viviana Job, Guy Schoehn, Ina Attrée, and Andréa Dessen

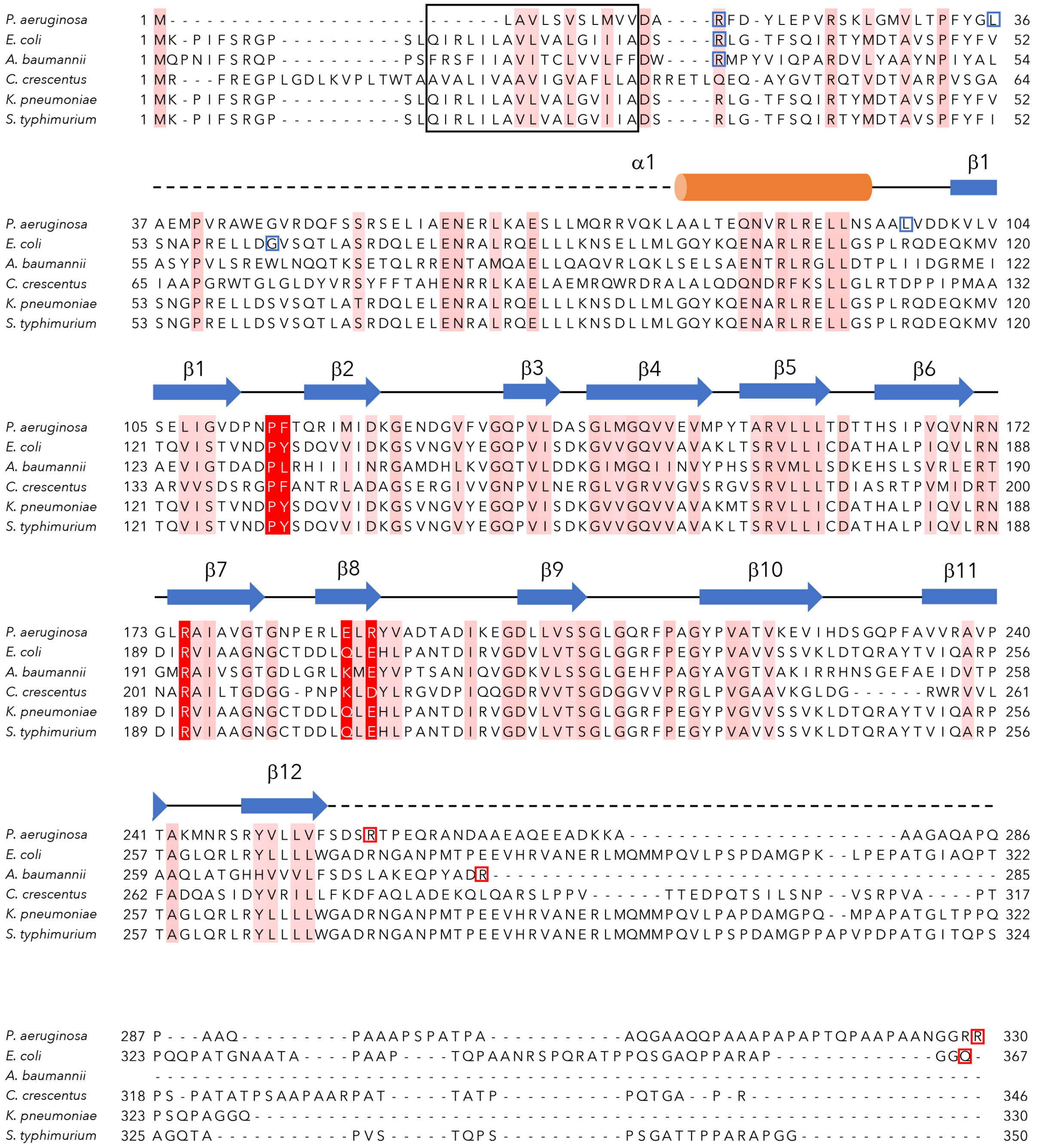


**Supplementary Figure 1**: **Structure-based sequence alignment of MreC sequences from common rod-shaped α- and γ- proteobacteria.** Blue and red squares indicate the N- and C-termini of MreC variants tested, respectively. Dark red highlights indicate residues of the three regions selected for mutagenesis in $MreC_{Pa}$. Light red highlights indicate highly conserved residues. The secondary structure elements indicated are those traced in the $MreC_{Pa}$ cryo-EM structure (residues 79 – 254). The initial black rectangle indicates the predicted transmembrane region. The Ala-Pro rich region in $MreC_{Pa}$ mentioned in the main text lies between residues 278 and 325.

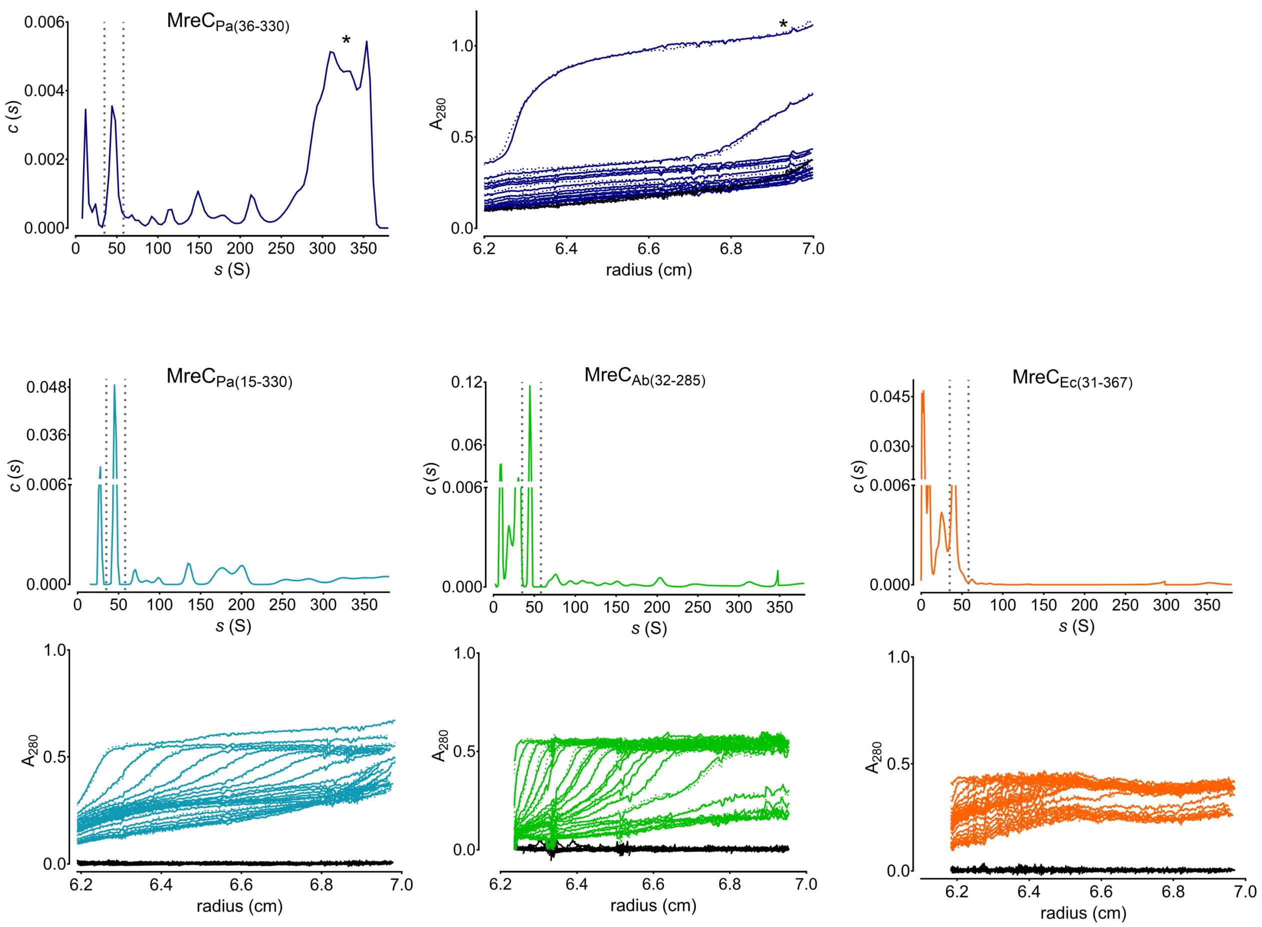


**Supplementary Figure 2: Details of SV-AUC experiments for MreC$_{Pa(15-330)}$, MreC$_{Pa(36-330)}$, MreC$_{Ec(31-367)}$, and MreC$_{Ab(32-285)}$.** Sedimentation velocity profiles were acquired for 14 h, with a rotor velocity of 62,000 g, at 20 °C and using absorbance monitoring at 280 nm. Protein samples were at 0.8 mg/ml. For each sample, results of the $c(s)$ analysis (from 0 to 380 $s$) and the residuals are shown. Solvent peaks at very small $s$ values were removed for clarity. MreC$_{Pa(36-330)}$ shows very fast-sedimenting species (*); the first scan displayed corresponds to 4 min of sedimentation, in accordance with the large species identified by EM. Vertical grey dots indicate the $c(s)$ regions of large oligomers common to all MreC forms studied in this work. Source data are provided as a Source Data file.

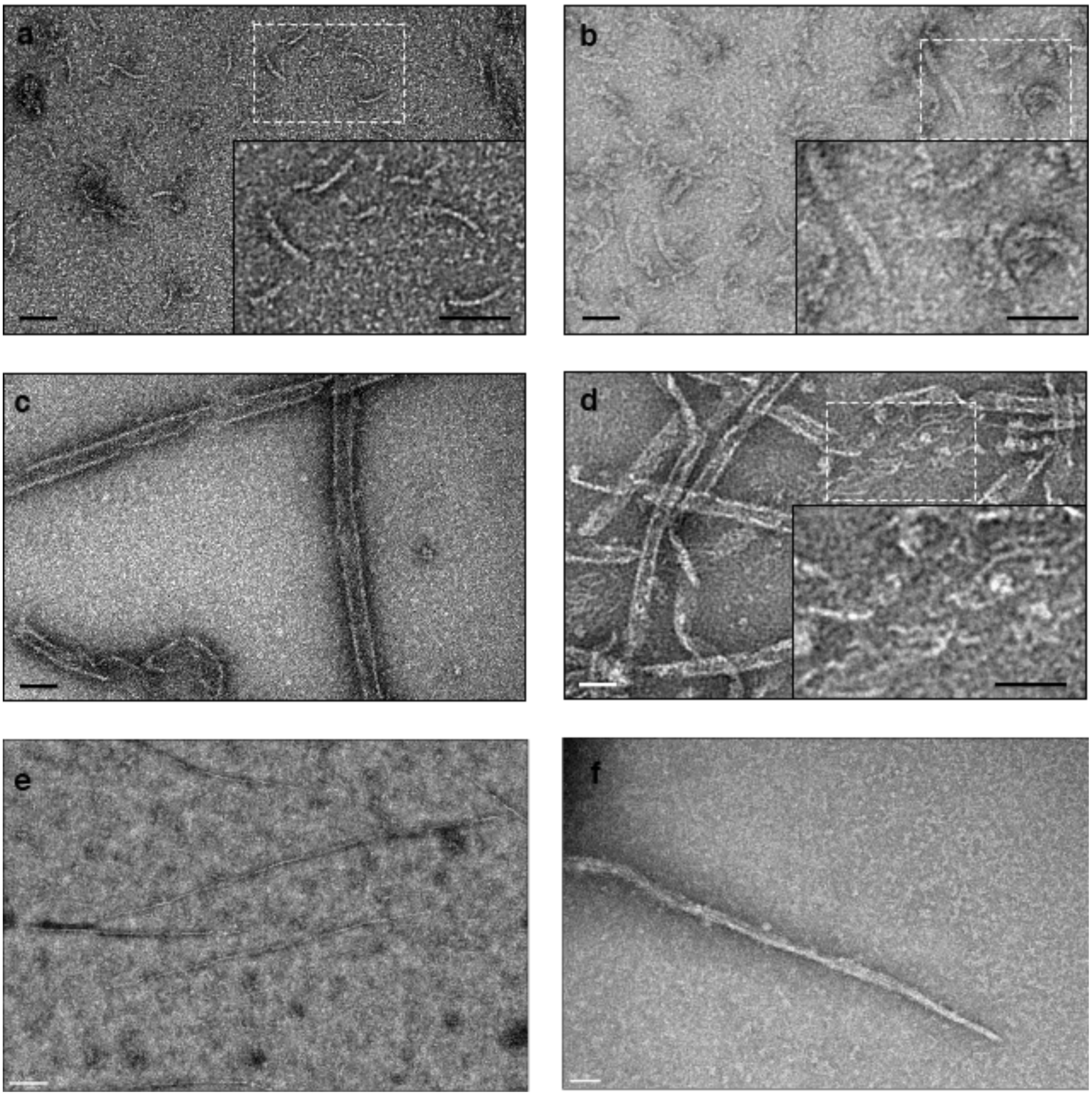


**Supplementary Figure 3**. **A gallery of MreC fibers, filaments, and patches visualized by negative stain EM in buffers at different pH values.** $MreC_{Pa}$ samples were visualized using uranyl acetate (Ur-Ace) as the EM dye unless otherwise noted. (a) $MreC_{Pa(36\text{-}330)}$ in Bis-tris pH 9.0, in many places resembling ‘beads on a string’, visualized by negative stain EM directly after gel filtration; (b) $MreC_{Pa(36\text{-}330)}$ in CHES pH 9.0, concentrated 2-3 X after gel filtration prior to EM; (c) $MreC_{Pa(36\text{-}330)}$ in CHES pH 9.0, concentrated 5 X after gel filtration prior to EM; (d) $MreC_{Pa(15\text{-}330)}$ in CHES pH 9.0, concentrated 5 X after gel filtration prior to EM. The latter form (starting at residue 15) showed a higher tendency to unwind than the more stable form, starting at residue 36. (e) $MreC_{Ab(32\text{-}285)}$ and (f) $MreC_{Ec(31\text{-}367)}$ in HEPES pH 7.5 formed thin filaments as well as smaller forms. In some instances, $MreC_{Ec(31\text{-}367)}$ filaments associated laterally, but did not generate ordered structures. Scale bars indicate 50 nm, with the exception of (e), that indicates 100 nm. For (a) and (b), over 50 images were made, from 5 different grids. For (c), at least 100 images were made, and 10 different grids prepared. For (d), over 70 images were prepared from 5 grids. For (e), 4 grids were prepared, generating over 40 images both in SST and Ur-Ace. For (f), 3 grids were prepared using SST, generating over 30 images.

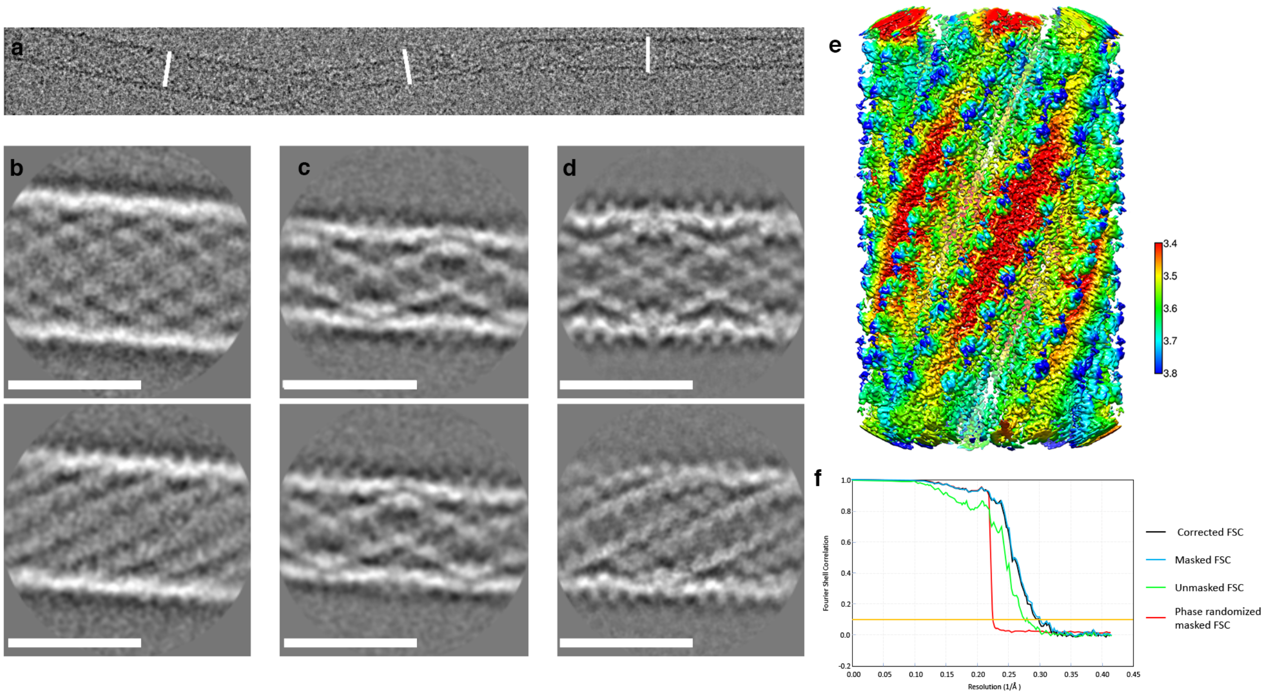


**Supplementary Figure 4: MreC$_{Pa(36\text{-}330)}$ tube morphology and class averages.** (a) Tube-like assemblies can have different diameters (220 Å on the left, 180 Å in the middle and 200 Å on the right). The vertical white scale bar represents 200 Å. (b, c, d) Class averages of segments of the 220 Å, 180 Å, 200 Å tubes. The white scale bar represents 200 Å. (e) Local resolution of the MreC$_{Pa(36\text{-}330)}$ density map, colored according to resolution as estimated by Resmap (Kucukelbir et al., 2014). (f) Fourier Shell Correlation (Chen et al., 2013) of the corrected (black), masked (blue), unmasked (green) and phase randomized (red) maps. The gold-standard Fourier shell correlation (FSC) of a masked map indicates a resolution of 3.5 Å for the cryo-EM map with the FSC = 0.143 criterium.

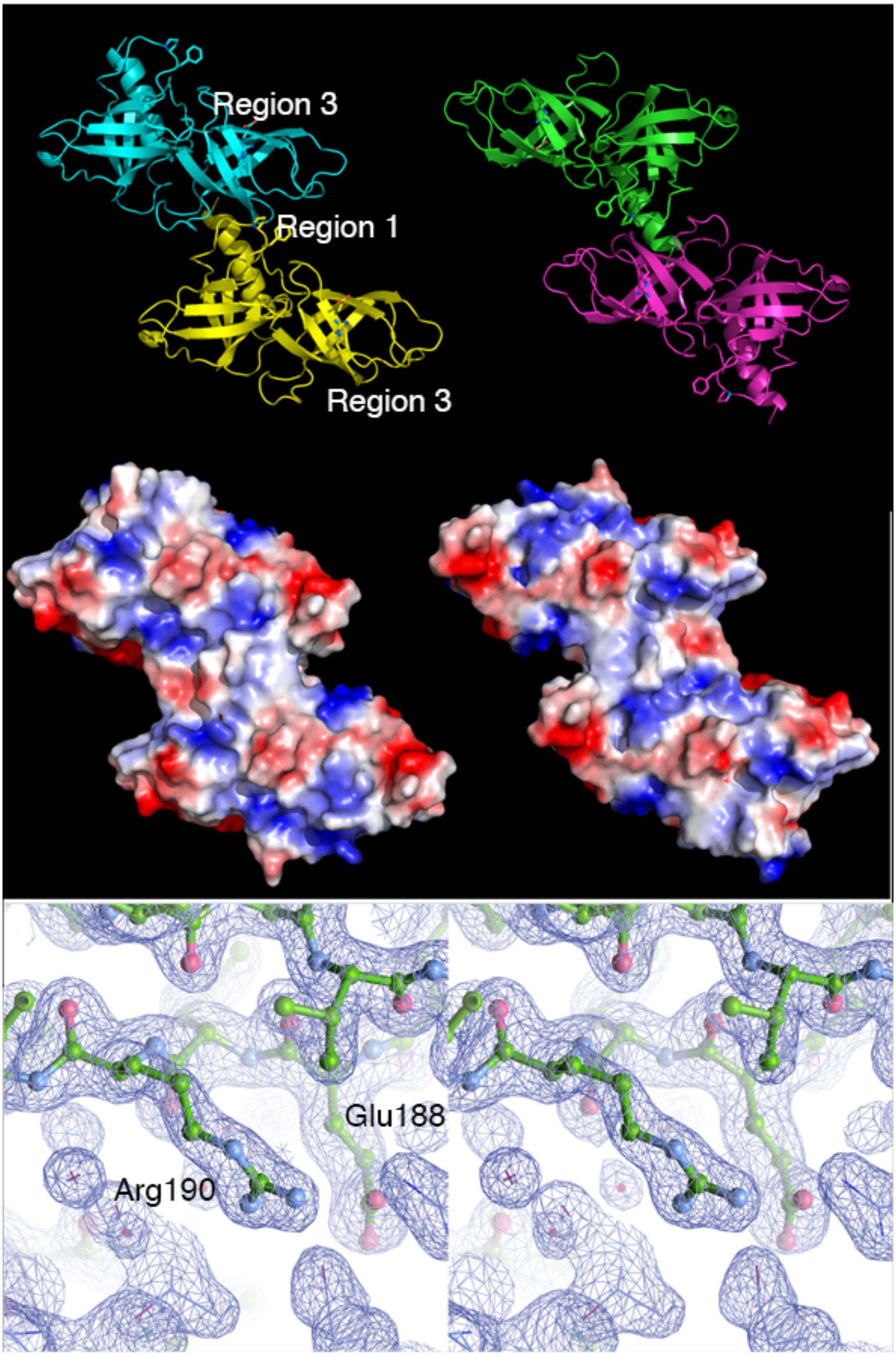


**Supplementary Figure 5: Surface potential representation of 'open' tetramers and 2Fo-Fc stereo map around region 3.** The two dimers that face each other to form the representative tetramer were separated and rotated, in order to display their interaction surfaces to the reader. Note that most of the charges are located in the 'β-sandwich' regions of $MreC_{Pa}$, with Region 3 playing a prominent role. The stereo map (lower panel), contoured at 1.28 rmsd, shows an interaction region between two monomers.

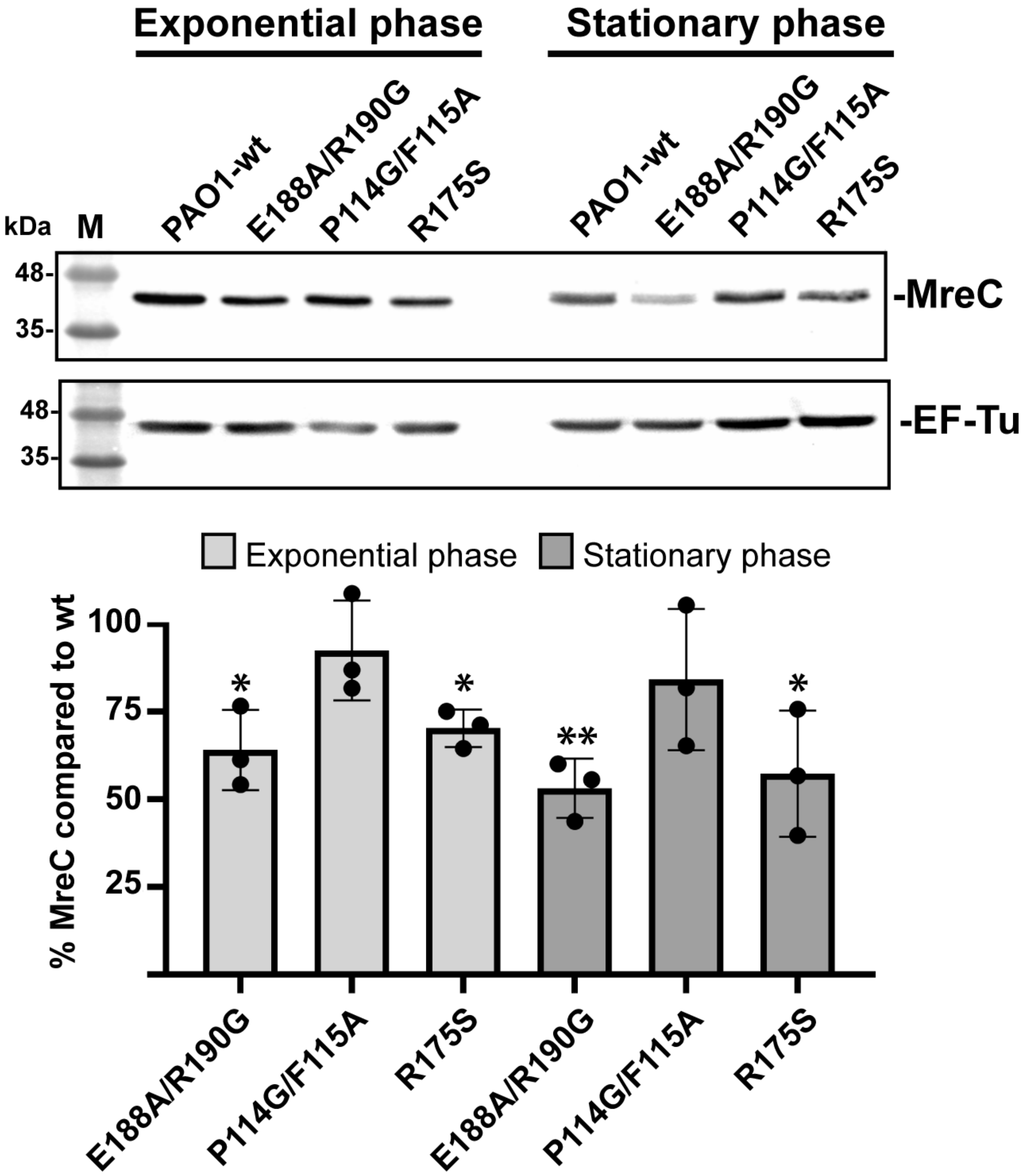


**Supplementary Figure 6. Mutations in MreC's Regions 2 and 3 affect MreC levels in both exponential and stationary phases**. All strains expressing wild-type or mutant MreC variants were grown to identical optical densities measured at 600 nm ($OD_{600}$) corresponding to exponential or stationary growth phases. MreC quantities were assessed using anti-MreC antibodies raised in rabbits by immunoblotting. One representative experiment is shown (of a total of 3; upper panel). MreC levels were first normalized to EF-Tu**,** and the amounts of mutant MreC were compared to those of the wild-type protein by analyzing samples loaded on the same gel. The mean values and standard deviations (SD) from three independent experiments (black dots) are shown. Statistical differences were evaluated using Tukey's multiple comparison test. p-values were (*p<0.05, **p<0.01) in stationary phase: p=0.008 (wt vs E188A/R190G), p=0.022 (wt vs R175S), and in exponential phase p=0.016 (wt vs E188A/R190G), p=0.026 (wt vs R175S) (lower panel). Source data are provided as a Source Data file.

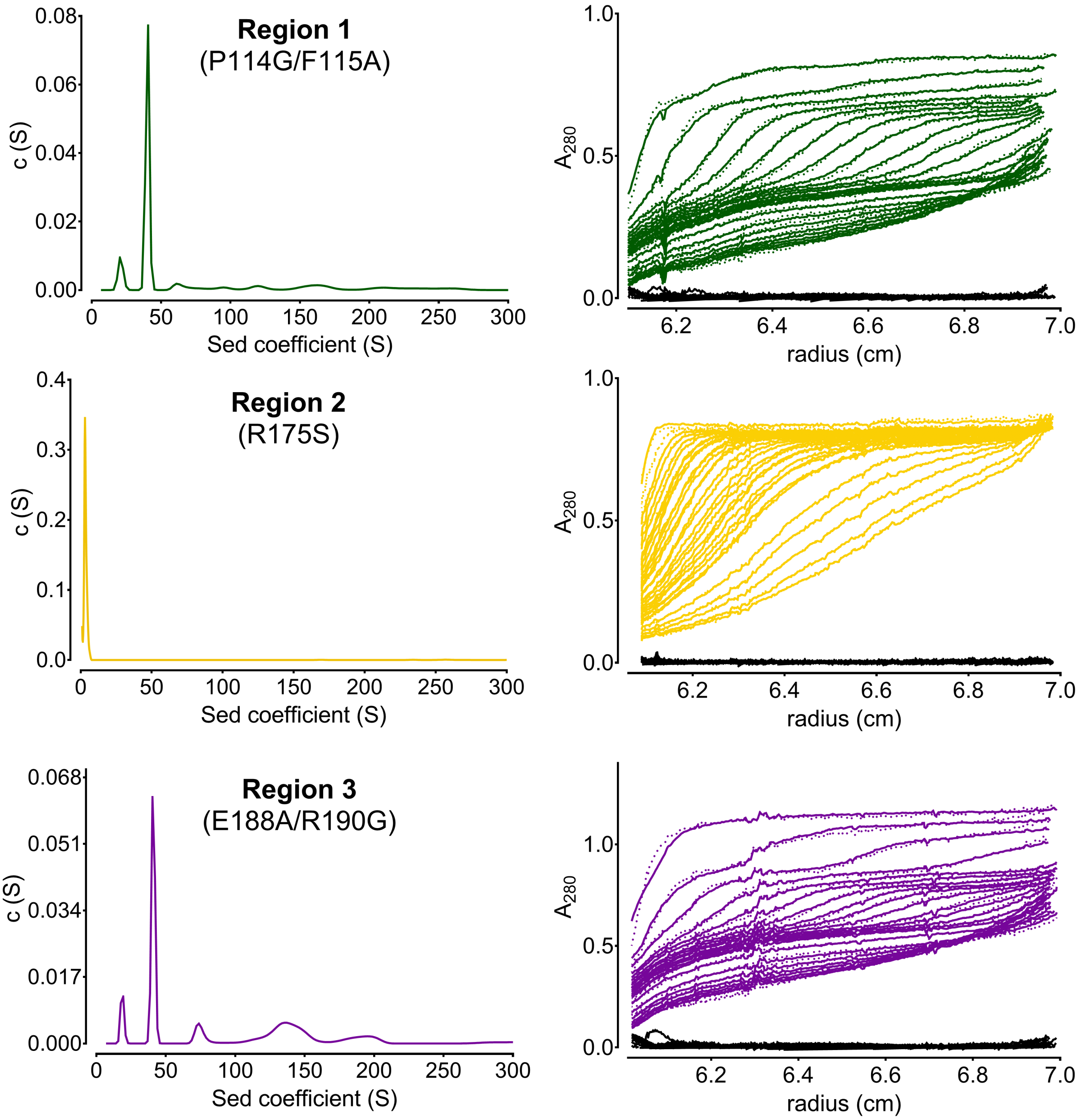


**Supplementary Figure 7**. Details of SV-AUC experiments for MreC$_{Pa(36\text{-}330)}$ variants carrying mutations in Regions 1, 2 and 3. Samples were at 0.8 mg/ml. Variants with mutations in Regions 1 and 3 mutants present high *s* values, while the Region 2 mutant has a sedimentation profile that is indicative of much smaller oligomers. The diagrams on the right show absorbance scans at 280 nm acquired during 14 h of sedimentation at 62,000 g and 20 °C. The black lines (inset) display the overlay of the residuals of the fit. Dotted lines represent the best-fit *c* (*s*) at different points in time. Solvent peaks at very small *s* values were removed for clarity. Source data are provided as a Source Data file.

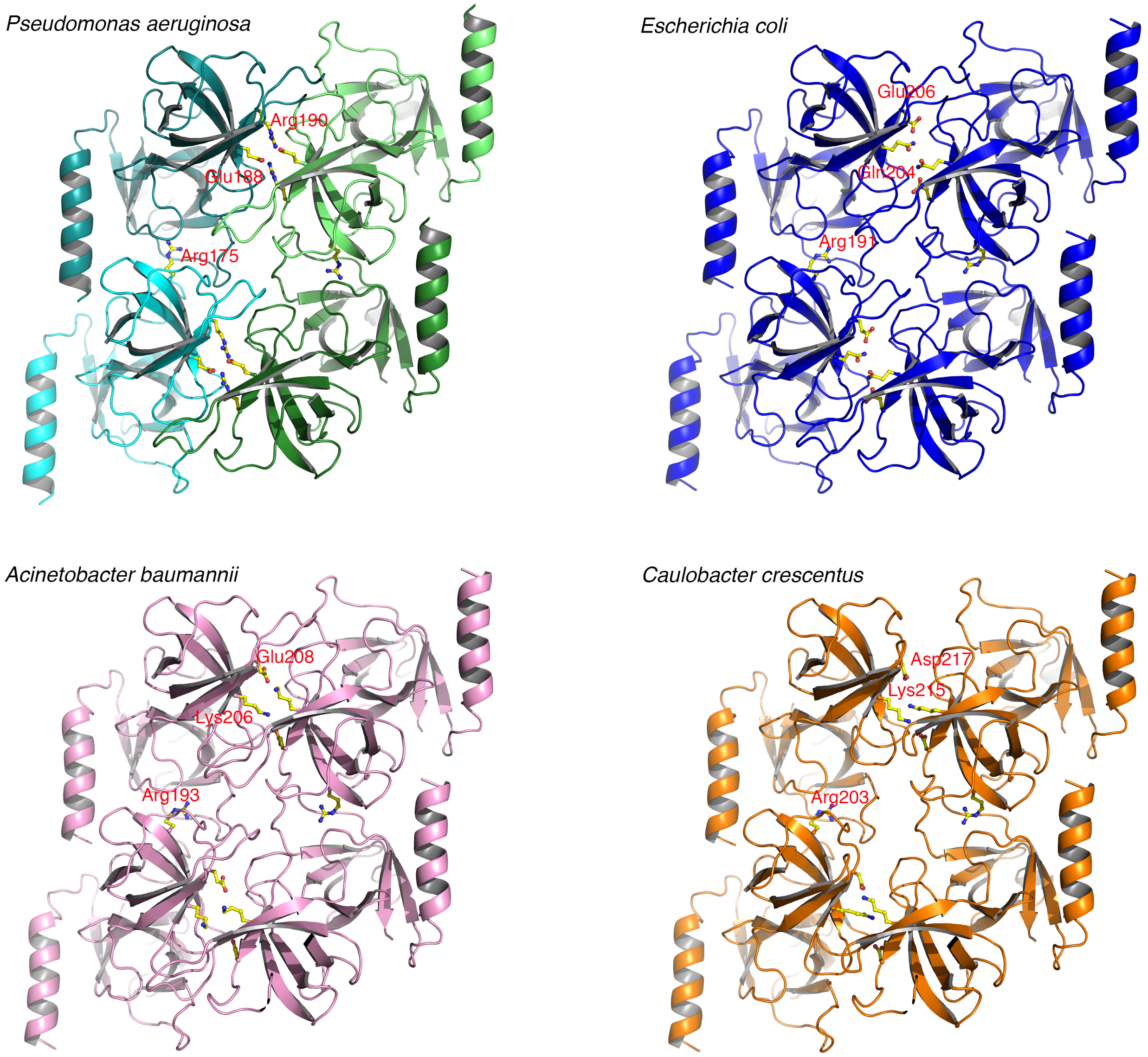


**Supplementary Figure 8. Gallery of MreC variants highlighting polar amino acids responsible for lateral interactions, and the conserved Arg that is key for longitudinal interactions**. Tetramers are shown in the same orientation, which is 180º away from the one displayed in Fig. 2. Apart from MreC from *P. aeruginosa*, whose structure was solved in this work, MreC tetramers from *E. coli*, *A. baumannii* and *C. crescentus* are models generated using the Swiss Model server. Note that all structures show key similarities at the interfaces. Residues are identified only once in each structure for simplicity.

**Supplementary Table 1: Cryo-EM data collection, refinement and validation statistics**

| | |
|---|---|
| **Data Collection** | |
| Microscope | Thermo Fisher Talos Glacios |
| Camera | Falcon II |
| Magnification | 120,000 (116 086) |
| Voltage (kV) | 200 |
| Number of frames | 20 |
| Electron exposure total ($e^-$/Å$^2$) | 43 |
| Defocus range (μm) | 1.0 – 3.5 |
| Pixel size (Å) | 1.206 |
| Symmetry imposed | Helical (62.998° twist, 10.3464 Å rise) and D2 |
| Initial/Final micrographs (no.) | 1200/739 |
| Final particles (no.) | 91840 |
| Map resolution (Å) 0.143 FSC | 3.5 |
| Map resolution range (Å) | 3.5-100 |
| **Refinement** | |
| Initial model used | $MreC_{Pa(97-258)}$ |
| Model resolution (Å) 0.5 FSC threshold | 3.8 |
| Map sharpening B factor (Å$^2$) | -189.492 |
| **Model composition**<br>Chains<br>Protein residues<br>ligands | <br>4<br>704<br>0 |
| B-factor (Å$^2$)<br>Protein min-max (mean) | <br>36.44-341.02 (127.12) |
| **R.m.s deviations**<br>Bond lengths (Å)<br>Bond angles (°) | <br>0.010<br>1.835 |
| **Validation**<br>MolProbity score<br>Clashscore | <br>2.40<br>18.63 |
| **Ramachandran plot**<br>Favored (%)<br>Allowed (%)<br>Disallowed (%) | <br>95.69<br>3.74<br>0.57 |

**Supplementary Table 2. Bacterial strains, plasmids and primers used in this work**

| | Genotype or relevant properties | Reference/Source |
|---|---|---|
| **Strains** | | |
| *P. aeruginosa* PAO1 | Wild-type reference strain (wound isolate) | 1, lab collection |
| *E. coli* TOP10 | Optimized for plasmid DNA transformation | Invitrogen |
| *E. coli* Mach1™-T1R | Optimized for plasmid DNA transformation | Invitrogen / ThermoFisher |
| *E. coli* BL21 (DE3) Gold | Used for protein expression strain | Novagen |
| **Plasmids** | | |
| pRK600 | Helper plasmid with conjugative properties ($Cm^R$) | 2 |
| pEXG2 | Allelic exchange vector ($Gm^R$), *sacB* | 3 |
| pUC57-mut-*mreC*-P114G/F115A | pUC57 carrying 397bp upstream flanking region, with sequences corresponding to P114G (ggc), F115A (gcc) and 396bp, downstream flanking region with *Eco*RI and *Hin*dIII restriction sites ($Amp^R$) | Genewiz |
| pEXG2-mut-*mreC*-P114G/F115A | pEXG2 carrying fragment P114G/F115A cloned with *Eco*RI and *Hin*dIII ($Gm^R$) | This work |
| pEXG2-*mreC1* | pEXG2 carrying DNA fragment from the *mreC* region obtained by SLIC ($Gm^R$) | This work |
| pEXG2-*mreC2* | pEXG2 carrying DNA fragment from the *mreC* region obtained by SLIC ($Gm^R$) | This work |
| pEXG2-mut-R175S | pEXG2 encoding part of $MreC^{R175S}$ ($Gm^R$) | This work |
| pEXG2-mut-E188A/R190G | pEXG2 encoding part of $MreC^{E188A/R190G}$ ($Gm^R$) | This work |
| pGEX-4T1 | Expression vector containing a N-terminal GST tag and a thrombin cleavage site for tag removal ($Ap^R$) | GE Healthcare |
| 17ABJRIC Ab MreC pMA-T | Plasmid harboring mreC from *Acinetobacter baumannii* used as template for construct amplification | Invitrogen / ThermoFisher |
| pGEX-PaMreC$_{(15\text{-}330)}$ | Expression of wild type MreC from *Pseudomonas aeruginosa* (residues 15 to 330) in fusion with a GST-tag ($Ap^R$) | This work |
| pGEX-PaMreC$_{(97\text{-}258)}$ | Expression of wild type MreC from *P. aeruginosa* (residues 97 to 258) in fusion with a GST-tag ($Ap^R$) | This work |
| pGEX-PaMreC$_{(36\text{-}330)\ wt}$ | Expression of wild type MreC from *P. aeruginosa* (residues 36 to 330) in fusion with a GST-tag ($Ap^R$) | This work |
| pGEX-PaMreC$_{(36\text{-}330)\ mut1}$ (P114G/F115A) | Expression of P114G/F115A MreC mutant from *P. aeruginosa* (residues 36 to 330) in fusion with a GST-tag ($Ap^R$) | This work |
| pGEX-PaMreC$_{(36\text{-}330)\ mut2}$ (R175S) | Expression of R175S MreC mutant from *P. aeruginosa* (residues 36 to 330) in fusion with a GST-tag ($Ap^R$) | This work |
| pGEX-PaMreC$_{(36\text{-}330)\ mut3}$ (E188A/R190G) | Expression of E188A/R190G MreC mutant from *P. aeruginosa* (residues 36 to 330) in fusion with a GST-tag ($Ap^R$) | This work |
| pGEX-AbMreC$_{(32\text{-}285)}$ | Expression of MreC from *Acinetobacter baumannii* (residues 32 to 285) in fusion with a GST-tag ($Ap^R$) | This work |

| | | |
|---|---|---|
| pGEX-EcMreC$_{(31\text{-}367)}$ | Expression of MreC from *Escherichia coli* (residues 31 to 367) in fusion with a GST-tag (Ap$^{R}$) | This work |

| **Primers** | **Sequence (5' > 3')** | **Use to create** |
|---|---|---|
| sR1-pEXG2-mut-R175S | ACCGAATTCGAGCTCGAGCCCCGATCTCGGTGAAGGTAGGC | pEXG2-*mreC1* |
| sF1-pEXG2-mut-R175S-E188A/R190G | GGTCGACTCTAGAGGATCCCCGGAGCGGATCATCAAACCGC | pEXG2-*mreC*1/2 |
| sR1-pEXG2-mut-E188A/R190G | ACCGAATTCGAGCTCGAGCCCGCAGCAGCAACACCCAGTAG | pEXG2-*mreC2* |
| F-mutagenesis-R175S-DdeI | AGGTCAATCGCAACGGCCTGAGCGCGATCGCCGTCGGCACCGGCAA | Site directed mutagenesis of pEXG2-*mreC1* to pEXG2-mut-R175S |
| R-mutagenesis-R175S-DdeI | TTGCCGGTGCCGACGGCGATCGCGCTCAGGCCGTTGCGATTGACCT | |
| F-mutagenesis-E188A/R190G-StyI | CGGCAATCCCGAGCGCCTGGCCTTGGGCTACGTCGCCGACACCGCC | Site directed mutagenesis of pEXG2-*mreC2* to pEXG2-mut-E188A/R190G |
| R-mutagenesis-E188A/R190G-StyI | GGCGGTGTCGGCGACGTAGCCCAAGGCCAGGCGCTCGGGATTGCCG | |
| F0-mut-mreC | CACTCCATGGACCTGCTCTC | PCR verification |
| R0-mut-mreC | TGACCATCCCGACGCGCTC | |
| MreC_5P | GGAGCGGATCATCAAACCGC | Mutant screening |
| MreC_3P | GCAGCATCGCCAGCCACAGC | |
| PaMreC F R15 BamHI | AAAGGATCCCGGTTCGACTATCTGGAGCCCGTCCG | pGEX-PaMreC$_{(15\text{-}330)}$ |
| PaMreC R stop XhoI | AAAACTCGAGTCAGCGGCGCCCCCCGTTCG | |
| PaMreC F L36 BamHI | GGTTCCGCGTGGATCCCTGGCGGAAATGCCGGTGC | pGEX-PaMreC$_{(36\text{-}330)\ wt}$ |
| PaMreC R stop XhoI | GATGCGGCCGCTCGAGTCAGCGGCGCCCCCCG | |
| F mutagenesis-T259/stop | CGTTGGCGCGCTGTTCCGGTCAGCGGCTGTCGCTGAACACC | First step of site directed mutagenesis to generate pGEX-PaMreC$_{(97\text{-}258)}$ |
| R mutagenesis-T259/stop | GGTGTTCAGCGACAGCCGCTGACCGGAACAGCGCGCCAACG | |
| Pa_MreC deltaHelix F | CTGGTCGACGACAAGGTAC | Second step of site directed mutagenesis to generate pGEX-PaMreC$_{(97\text{-}258)}$ |
| Pa_MreC deltaHelix R | GGATCCACGCGGAACCAG | |
| PaMreC P114G+F115A R | ACGCCGATCAGCTCGCTG | site directed mutagenesis to generate pGEX-PaMreC$_{(36\text{-}330)}$ mut 1 (P114G/F115A) |
| PaMreC P114G+F115A F | CGATCCGAACGGCGCGACCCAACGCATCATGATCGACAAG | |
| PaMreC R175S R | CGATTGACCTGCACCGGGATAC | site directed mutagenesis to generate pGEX-PaMreC$_{(36\text{-}330)}$ mut 2 (R175S) |
| PaMreC R175S F | CAACGGCCTGAGCGCCATCGC | |
| PaMreC E188A+R190G F | GAGCGCCTGGCATTGGGCTACGTCGCCG | site directed mutagenesis to generate pGEX-PaMreC$_{(36\text{-}330)}$ mut 3 (E188A/R190G) |
| PaMreC E188A+R190G R | GGGATTGCCGGTGCCGACG | |
| BamHI Ab_MreC R32 F | AAGGATCCCGTATGCCGTATGTTATTCAGCCTGC | pGEX-AbMreC$_{(32\text{-}285)}$ |
| XhoI stop AbMreC R | TTCTCGAGTTAACGATCTGCATACGGCTGTTCTTTTGC | |
| BamHI Ec_MreC R31 F | AAAAGGATCCCGCCTGGGGACGTTCAGTCAAATC | pGEX-EcMreC$_{(31\text{-}367)}$ |
| XhoI stop Ec_MreC R | AATACTCGAGCTATTGCCCTCCCGGCGCACGC | |

## SUPPLEMENTARY REFERENCES


Chen, S., McMullan, G., Faruqi, A.R., Murshudov, G.N., Short, J.M., Scheres, S.H.W., and Henderson, R. (2013) High-resolution noise substitution to measure overfitting and validate resolution in 3D structure determination by single particle electron cryomicroscopy. Ultramicroscopy 135, 24-35.

Holloway, BW (1955) Genetic recombination in Pseudomonas aeruginosa. J. Gen. Microbiol. 13, 572-581.

Kessler, B., de Lorenzo, V. and Timmis, K.N. (1992) A general system to integrate lacZ fusions into the chromosomes of gram-negative eubacteria: regulation of the Pm promoter of the TOL plasmid studied with all controlling elements in monocopy. Mol. Gen. Genetics MGG 233, 293-301.

Kucukelbir, A., Sigworth, F.J., and Tagare, H.D. (2014) Quantifying the local resolution of cryo-EM density maps. Nature Meth 11, 63-65.

Rietsch, A., Vallet-Gely, I., Dove, S.L. and Mekalanos, J.J. (2005) ExsE, a secreted regulator of type III secretion genes in Pseudomonas aeruginosa. Proc Natl Acad Sci U S A, 102, 8006-8011.
