# Supplementary material for: Self-association of MreC as a regulatory signal in bacterial cell wall elongation
Source: Nat Commun. 2021 May 20;12:2987. doi: 10.1038/s41467-021-22957-9 (PMC8137920; doi:10.1038/s41467-021-22957-9)
Supplement: Supplementary file 3 — Reporting Summary [file 41467_2021_22957_MOESM3_ESM.pdf]

nature research

Corresponding author(s): Andrea Dessen

Last updated by author(s): Feb 28, 2021

Reporting Summary

Nature Research wishes to improve the reproducibility of the work that we publish. This form provides structure for consistency and transparency in reporting. For further information on Nature Research policies, see our Editorial Policies and the Editorial Policy Checklist.

Statistics

For all statistical analyses, confirm that the following items are present in the figure legend, table legend, main text, or Methods section.

n/a	Confirmed	
☐	☒	The exact sample size (*n*) for each experimental group/condition, given as a discrete number and unit of measurement
☐	☒	A statement on whether measurements were taken from distinct samples or whether the same sample was measured repeatedly
☐	☒	The statistical test(s) used AND whether they are one- or two-sided<br>*Only common tests should be described solely by name; describe more complex techniques in the Methods section.*
☒	☐	A description of all covariates tested
☒	☐	A description of any assumptions or corrections, such as tests of normality and adjustment for multiple comparisons
☒	☐	A full description of the statistical parameters including central tendency (e.g. means) or other basic estimates (e.g. regression coefficient) AND variation (e.g. standard deviation) or associated estimates of uncertainty (e.g. confidence intervals)
☒	☐	For null hypothesis testing, the test statistic (e.g. *F*, *t*, *r*) with confidence intervals, effect sizes, degrees of freedom and *P* value noted<br>*Give P values as exact values whenever suitable.*
☒	☐	For Bayesian analysis, information on the choice of priors and Markov chain Monte Carlo settings
☒	☐	For hierarchical and complex designs, identification of the appropriate level for tests and full reporting of outcomes
☒	☐	Estimates of effect sizes (e.g. Cohen's *d*, Pearson's *r*), indicating how they were calculated

Our web collection on statistics for biologists contains articles on many of the points above.

Software and code

Policy information about availability of computer code

Data collection	cryo-EM: FEI EPU 1.9<br>X-ray crystallography: LNLS Synchrotron (Campinas), beamline MX-2, with optics controlled by EPICS and with a Pilatus 2M detector. System controlled by MXCube.<br>MreC sequences: Uniprot.org
Data analysis	cryo-EM: MotionCor2 2.1.6, Gctf 1.06, Relion 3.0.8 (as provided by SBgrid)<br>X-ray crystallography: XDS package (Jan 9, 2019), ADXV (1.9.12), XDSGUI (2018), STARANISO (web server), CCP4-7.0 suite, Pymol 1.7<br>Phylogenetic analysis: PROMALS3D, MEGA X, iTOL (v5)<br>ImageLab (BioRad, version 6.0.1).<br>SigmaPlot (Ritme, version 11.0)<br>Prism (GraphPad, version 7.04)

For manuscripts utilizing custom algorithms or software that are central to the research but not yet described in published literature, software must be made available to editors and reviewers. We strongly encourage code deposition in a community repository (e.g. GitHub). See the Nature Research guidelines for submitting code & software for further information.

Data

Policy information about availability of data

All manuscripts must include a data availability statement. This statement should provide the following information, where applicable:

- Accession codes, unique identifiers, or web links for publicly available datasets
- A list of figures that have associated raw data
- A description of any restrictions on data availability

Accession codes listed for both crystallography and cryo-EM data (PDB and EMDB): 6ZLV, 6ZM0, and EMD-11275. No restrictions for any data described in the manuscript.

Field-specific reporting

Please select the one below that is the best fit for your research. If you are not sure, read the appropriate sections before making your selection.

☒ Life sciences ☐ Behavioural & social sciences ☐ Ecological, evolutionary & environmental sciences

For a reference copy of the document with all sections, see nature.com/documents/nr-reporting-summary-flat.pdf

Life sciences study design

All studies must disclose on these points even when the disclosure is negative.

Sample size	Synchrotron X-ray diffraction data correspond to 3,600 images with a oscillation steps of 0.1 degrees in order to allow for collection of a full data set.
Data exclusions	Crystallography: no reflections were excluded. Resolution limits were determined by STARANISO (best possible resolution).<br>Cryo-EM: particle sorting and exclusion in RELION followed standard procedures and the remaining particle numbers are mentioned in the methods section.
Replication	Negative staining: several replicates; cryo-EM grid: 4 grids were frozen; data collection performed on one<br>Microbiology : 3 replicates
Randomization	Mutant MreC cell lines were chosen randomly.
Blinding	N/A

Reporting for specific materials, systems and methods

We require information from authors about some types of materials, experimental systems and methods used in many studies. Here, indicate whether each material, system or method listed is relevant to your study. If you are not sure if a list item applies to your research, read the appropriate section before selecting a response.

Materials & experimental systems

n/a	Involved in the study	
☐	☒	Antibodies
☒	☐	Eukaryotic cell lines
☒	☐	Palaeontology and archaeology
☒	☐	Animals and other organisms
☒	☐	Human research participants
☒	☐	Clinical data
☒	☐	Dual use research of concern

Methods

n/a	Involved in the study	
☒	☐	ChIP-seq
☒	☐	Flow cytometry
☒	☐	MRI-based neuroimaging

Antibodies

Antibodies used	Polyclonal serum raised against MreC-Pa (Biotem), EF-Tu (Hycult Biotech #HM6010, 1: 10,000 dilution), secondary antibodies : anti-rabbit-HRP (Sigma #A9169; 1: 20,000 dilution) and anti-mouse-HRP (Sigma #A9044; 1: 20,000 dilution)
Validation	Polyclonal anti-MreC serum obtained from rabbits was validated by comparing the wild-type P. aeruginosa strain and the mutant strain harboring MreC of different sizes. The unique band was recognized on Western blots using whole cell extracts. The anti-EF-Tu antibody was used as recommended by the manufacturer (https://www.hycultbiotech.com/hm6010).<br>Secondary antibodies were used as recommended by the manufacturer (SIGMA, https://www.sigmaaldrich.com/catalog/product/sigma/a9169?lang=fr&region=FR; https://www.sigmaaldrich.com/catalog/product/sigma/a9044?lang=fr&region=FR).
